# Co-occurrence of OXA-232, RmtF-encoding plasmids, and pLVPK-like virulence plasmid contributed to the generation of ST15-KL112 hypervirulent multidrug-resistant *Klebsiella pneumoniae*

**DOI:** 10.3389/fmicb.2023.1133590

**Published:** 2023-02-28

**Authors:** Chunyang Wu, Ying Zhou, Wenxiu Ai, Yinjuan Guo, Xiaocui Wu, Bingjie Wang, Huilin Zhao, Lulin Rao, Xinyi Wang, Jiao Zhang, Fangyou Yu, Liangxing Wang

**Affiliations:** ^1^Department of Respiratory Medicine, The First Affiliated Hospital of Wenzhou Medical University, Wenzhou, China; ^2^Department of Clinical Laboratory Medicine, Shanghai Pulmonary Hospital, Tongji University School of Medicine, Shanghai, China; ^3^Xiangyang Central Hospital, Affiliated Hospital of Hubei University of Arts and Science, Xiangyang, China; ^4^Department of Laboratory Medicine, The First Affiliated Hospital of Wenzhou Medical University, Wenzhou, China

**Keywords:** *bla*_OXA-232_, *rmtF*, *Klebsiella pneumoniae*, carbapenemase, mobile element, plasmid

## Abstract

The emergence of carbapenem-resistant *Klebsiella pneumoniae* (CRKP) strains and restricted therapeutic options pose a global threat to public health. Aminoglycosides are a wise choice, which can effectively reduce the mortality rate when combined with β-lactam drugs. However, in this study, we identified a ST15-KL112 CRKP FK3006 which not only exhibited resistance to carbapenems, but also exhibited high level resistance to aminoglycosides. In addition to the multidrug resistant phenotype, FK3006 also owned typical pathogenic characteristic, including hypermucoviscosity and hypervirulence phenotypes. According to the whole-genome sequencing, one pLVPK-like virulence plasmid, and three key resistant plasmids (*bla*_OXA-232_, *bla*_CTX-M-15_, and *rmtF*) were observed in FK3006. Compared to other typical ST15 CRKP, the presence of pLVPK-like virulence plasmid (p3006-2) endowed the FK3006 with high virulence features. High siderophore production, more cell invasive and more resistant to serum killing was observed in FK3006. The *Galleria mellonella* infection model also further confirmed the hypervirulent phenotype of FK3006 *in vivo.* Moreover, according to the conjugation assay, p3006-2 virulence plasmid also could be induced transfer with the help of conjugative IncFII_K_ p3006-11 plasmid (*bla*_CTX-M-15_). In addition to the transmissible plasmid, several insertion sequences and transposons were found around *bla*_CTX-M-15_, and *rmtF* to generate the mobile antimicrobial resistance island (ARI), which also make a significant contribution to the dissemination of resistant determinants. Overall, we reported the uncommon co-existence of *bla*_OXA-232_, *rmtF*-encoding plasmids, and pLVPK-like virulence plasmid in ST15-KL112 *K. pneumoniae*. The dissemination threatens of these high-risk elements in *K. pneumoniae* indicated that future studies are necessary to evaluate the prevalence of such isolates.

## Introduction

The emergence of carbapenem-resistant *Klebsiella pneumoniae* (CRKP) has become a major challenge facing clinical management and global public health, because of the extremely limited antibiotic therapy options (Ernst et al., 2020). Aminoglycosides are important options for treating infections caused by CRKP and are generally administered in combination with β-lactam agents and tigecycline (Daikos et al., 2014; Karaiskos et al., 2019). However, increasing rates of aminoglycoside resistance in CRKP have been reported in recent years, posing a new challenge for treatment (Galani et al., 2019). Hence, verifying the related mechanism and demonstrating the potential of the spread of these resistant phenotypes in clinical isolate are vital clues to solving antibiotic resistance.

It has been highlighted *Klebsiella pneumoniae* carbapenamase (KPC) is the most prevalent in China, it is noteworthy that OXA-48-like carbapenemases are common carbapenemases in *Enterobacterales* in certain regions of the world (Pitout et al., 2019). To date, several variants that differ from OXA-48 by only a few amino acids and display similar enzymatic profiles with OXA-48 have been identified. OXA-232 differs from OXA-48 by five amino acid substitutions, exhibiting a lower ability to hydrolyze carbapenems but greater hydrolytic activities against penicillin than OXA-48 (Potron et al., 2013; Miltgen et al., 2020). Since the first report of an OXA-232-producing *K. pneumoniae* strain in China in 2016, such isolates has became epidemic in China, and usually associated with a clonal dissemination of ST15 *K. pneumoniae* (Yin et al., 2017; Wang et al., 2022). Most aminoglycoside resistance mechanisms were associated with the aminoglycoside-modifying enzymes, among which only 16S rRNA methyltransferase (16S-RMTase)-encoding genes could mediate high-level resistance to aminoglycosides (Ramirez and Tolmasky, 2010). Among these genes, *rmtB* and *armA* present the most widespread 16S rRNA methylase genes, with *rmtF* is rarely reported (Nagasawa et al., 2014). Notably, these antibiotic resistances could be carried by various mobile genetic elements (MGEs), once these resistant elements co-existence in one host, the therapeutic options would be very limited.

*Klebsiella pneumoniae* has an exceptional ability to acquire exogenous resistance-encoding and hypervirulence-encoding genetic elements. For a long period, *K. pneumoniae* did not simultaneously encode the phenotypes of multidrug-resistance (MDR) and hypervirulence. However, carbapenem-resistant hypervirulent *K. pneumoniae* (hv-CRKP) isolates have been increasingly reported in recent years. The epidemic of hv-CRKP strains has emerged as a worldwide public health concern as they may cause untreatable, severe infections (Yang et al., 2021).

Although the hv-CRKP has increased rapidly, most were associated with the KPC carbapenemases and ST11 *K. pneumoniae*, the ST15-OXA-232 hv-CRKP was uncommon. In this study, we identified a hypermucovious multidrug-resistant ST15 *K. pneumoniae* (FK3006), exhibiting resistance to both aminoglycosides and ertapenem. Then, we applied whole-genome-sequencing (WGS) to explore the potential molecular mechanisms and observed four key plasmids. We also applied the conjugation assay to further determine the dissemination of these high-risk determinants and verified the relevant virulence phenotype of FK3006. In addition to the plasmids, we described other related MEGs through genetic comparisons as well. Overall, our goal was to report and describe a clinical hypervirulent carbapenem-resistant *K. pneumoniae* clearly, and emphasize the possible risk of these strains.

## Materials and methods

### Bacterial isolates

First, the 207 CRKP isolates from patients at 12 tertiary China teaching hospitals in eight Chinese provinces were collected From January 2015 to May 2021. And then the detached samples were cultured on blood agar plate and identified by MALDI-TOF MS. FK3006 was isolated from the sputum sample, for it is the only isolate harboring the co-existence of *bla*_OXA-232_, *bla*_CTX-M-15_, *rmtF*, and virulence plasmid analyzed by WGS. *K. pneumoniae* strain 3036 (FK3036) was used as control strain for the virulence-negative resistant strain (ST15, none Virulence factors) and NUTH-K2044(ST23) was used as a virulence-positive control (Supplementary Table S1). Plasmid conjugation was performed with *Escherichia coli* 600(EC600, rifampicin-resistant), which was used as the recipient strain.

### Antimicrobial susceptibility test

The MICs of the FK3006, EC600, and transconjugants (FK3006JH-1, FK3006JH-2) were determined by standard broth microdilution method following the Clinical and Laboratory Standards Institute guidelines (CLSI, 2020), except for colistin and tigecycline, for which the European Committee on Antimicrobial Susceptibility Testing (EUCAST) breakpoints were used (EUCAST, 2020). Each AST was independently repeated three times in our study. *Escherichia coli* ATCC 25922 was used as the quality control organism in MIC determination.

### Conjugation assay

The horizontal transferability of *bla*_OXA-232_, *bla*_CTX-M-15_, *rmtF*, and *iucA* were examined using the conjugation assay with *E. coli* 600. The FK3006 was used as donor strain, and *E. coli* 600 was used as the recipient strain. The recipients and donors were cultured in Luria-Bertani broth (37°C) until logarithmic phase (OD600 = 0.4–0.6), mixed in a ratio of 2:1 (200 μl,100uL) in 4 ml LB broth for 24 h. And then the serial dilutions were plated on selective media with appropriate antibiotics (gentamicin, 8 μg/ml[*rmtF*]; ampicillin, 100 μg/ml[*bla*_CTX-M-15_]; meropenem, 1 μg/ml[*bla*_OXA-232_]; dipotassium tellurite, 8 μg/ml[*iucA*]; rifampicin, 600 μg/ml). The frequency of plasmid transfer was calculated as the number of transconjugants per recipient. The transconjugants acquiring gene were confirmed by PCR and primers are listed in Supplementary Table S2.

### Growth assays to assess in fitness

After the plasmid was confirmed to be obtained, we applied growth curve assays to investigate fitness. Transconjugants (FK3006JH-1 and FK3006JH-2) and EC600 were cultured in LB medium overnight, then diluted to an OD600 of 0.01 and grown at 37°C for 24 h. Culture densities were determined by measuring the OD600 every 1 h for the first 12 h and then 24 h (Liu et al., 2016).

### Whole genome sequencing and bioinformatics analysis

Bacterial genomic DNA of FK3006 was isolated using the Qiagen DNA extraction Kit (Qiagen, Germany) and the genome sequencing was then performed by the PacBio Sequel platform and the Illumina NovaSeq 6,000 platform. CANU (version 1.7.1) software was applied to assemble the data acquired by PacBio platform sequencing. The ORF prediction was measured in SnapGene (version 4.2.4). Resistant plasmid replicons were predicted using the PlasmidFinder tool^1^. To verify whether the plasmid was also a conjugative plasmid, VRprofile^2^ and OriT Finder website^3^ were performed to analysis of the four conjugal modules in the plasmid, including the relaxase gene, the origin of transfer site (oriT), the type IV secretion system gene cluster (T4SS), and the type IV coupling protein (T4CP) gene. The transposons (Tns) and insertion sequences (ISs) were also annotated and determined through the VRprofile and ISFinder^4^. We used BLAST Ring Image Generator (BRIG) to determine similar plasmids by comparing their identities and coverages. The circular representation was performed by Proksee^5^. The gene environments surrounding the antibiotic resistance gene was analyzed by Easyfig software. VFDB^6^ was performed to annotate the virulence factors.

### Quantitative siderophore production assay

Briefly, bacterial clones were diluted with saline to a concentration of approximately 10^8^ CFU/ml, and then 1 μl bacterial clones were dropped on CAS and King’s B (2:1) plates. After incubation for 24 h at 37°C, siderophore production was determined by the presence of an orange halo around the bacterial colonies (Zhou et al., 2022).

### *Galleria mellonella* killing studies

Caterpillars of *Galleria mellonella* were stored at 4°C before use for ensuring their health and fitness. Caterpillars were selected weighing 200–250 mg each for the study. For the FK3006, EC600, FK3006JH-1 and FK3006JH-2 groups, the caterpillars were injected with 10 μl (~1 × 10^6^ CFU) bacterial suspension at the left proleg, while the control groups were injected with PBS or empty syringe. A minimum of 10 caterpillars was used in each treatment group; they then were kept in culture at 37°C and inspected for 144 h. The survival rates of each group were recorded for each day (Li et al., 2020).

### Serum killing assay

Briefly, serum was separated from blood samples and stored at-80°C. An inoculum of 25 μl (~1 × 10^6^ CFU) bacteria prepared from the mid-log phase was incubated with 75 μl pooled human sera. The mixture was taken at 0, 1, 2, and 3 h, and then incubated on the MHA plate. The numbers of viable bacteria were determined at 24 h (Liu et al., 2019).

### Infection of human cells and *Klebsiella pneumaniae*-mediated cytotoxicity

Approximately 1 × 10^5^ A549 human lung epithelial cells (ATCC CCL-185) were grown in each well of 24-well plates in DMEM medium containing 10% fetal calf serum at 37°C with 5% CO_2_ for 12 h and then incubated for 21 h at 37°C with 2 × 10^7^ CFU bacteria (Gottig et al., 2016). After centrifugation (3,000 rpm, 5 min, 4°C), the LDH in the supernatant was measured using the LDH Cytotoxicity Assay kit according to the instructions (Solarbio BC0685).

### S1 pulsed-field gel electrophoresis

The S1-Nuclease Pulsed Field gel electrophoresis (S1-PFGE) was used to determine the existence of plasmids profiles in donor strains, and recipient strains. In short, the isolates were embedded in 1% Seakem Gold agarose and digested with S1 nuclease (Takara, Dalian, China). PFGE analysis was carried out using a CHEF-Mapper system (Bio-Rad) for 19 h with a switch time 4.0–40 s (parameters: 14°C, voltage 6 V/cm, and electric field angle 120°). The *X*baI digested DNA of Salmonella serotype Braenderup H9812 was considered as a molecular size marker (Ai et al., 2021).

### Statistical analysis

Data analyses were executed with the software GraphPad Prism 8.0.2. Results are shown as a two-tailed non-parametric Student’s test. For *in vivo* and vitro experiments survival data were analyzed by the Log Rank test (Mantel-Cox). *P < 0.05* was considered to be statistically significant.

### Nucleotide accession number

The complete nucleotide sequences of the chromosome and p3006-2, p3006-3, p3006-7, and p3006-11 plasmid were deposited as GenBank accession numbers JANCTJ010000000 consists of sequences JANCTJ010000001-JANCTJ010000014.

## Results

### FK3006 was a multidrug-resistant strain

To characterize the antibiotic-resistant phenotype of FK3006, the 18 antibiotics susceptibility was tested in this strain. Results showed that FK3006 was a multi-drug resistant strain, which not only exhibited high-level resistance to both aminoglycoside antibiotics and a series of β-lactam antibiotics, but also was resistant to ciprofloxacin, sulfamethoxazole, minocycline, and piperacillin-tazobactam (Table 1). Although it was sensitive to imipenem, it still exhibited low-level resistance to other carbapenems including meropenem and ertapenem.

**Table 1:** Antimicrobial drug susceptibility profiles.

Drug class	Antibiotics	MIC (mg/L)/antimicrobial susceptibility							
		FK3006	S/I/R	EC600	S/I/R	FK3006JH-1	S/I/R	FK3006JH-2	S/I/R
Carbapenems	Ipm	<=0.5	S	<=0.5	S	<=0.5	S	<=0.5	S
	Etp	1	I	<=0.5	S	<=0.5	S	<=0.5	S
	Mer	2	I	<=0.5	S	<=0.5	S	<=0.5	S
β-Lactam/β-lactamase	P/T	>128/4	R	<=4/4	S	<=4/4	S	<=4/4	S
Inhibitor complexes	Caz/Avi	<=0.25/4	S	<=0.25/4	S	<=0.25/4	S	<=0.25/4	S
Monocyclic β-lactam	Azt	>32	R	<=1	S	>32	R	>32	R
Cephalosporin	Fox	32	R	4	S	4	S	2	S
	Ctx	>64	R	<=1	S	>64	R	>64	R
	Cpe	>16	R	<=0.5	S	>16	R	>16	R
	Caz	>32	R	<=1	S	>32	R	>32	R
Fluoroquinolones	Cip	>4	R	<=0.25	S	0.5	I	0.5	I
Folate metabolic pathway	CoSMZ	>2/38	R	<=0.5/9.5	S	>2/38	R	>2/38	R
Inhibitors	Te	8	I	<=1	S	<=1	S	<=1	S
Tetracyclines	Min	>8	R	<=2	S	<=2	S	<=2	S
	TGC	1	S	<=0.25	S	<=0.25	S	<=0.25	S
Polymyxin B	PB	1	I	1	S	1	S	1	S
Aminoglycosides	Gen	16	R	<=0.5	S	<=0.5	S	<=0.5	S
	AMK	>64	R	<=2	S	<=2	S	<=2	S

MIC, minimum inhibitory concentration; S, susceptible; I, intermediate; R, resistant; Ipm, imipenem; Etp, Ertapenem; Mer, meropenem; P/T, piperacillin–tazobactam; Caz/Avi, ceftazidime–avibactam; Azt, aztreonam; Min, minocycline; Cpe, cefepime; Ctx, cefotaxime; Caz, ceftazidime; Fox, cefoxitin; Cip, ciprofloxacin; Te, tetracycline; CoSMZ, sulfamethoxazole; TGC, tigecycline; PB, polymyxin B; Gen, gentamicin; AMK, amikacin.

### FK3006 co-harboring multiple resistance and virulence determinants

According to the subsequent WGS-based analysis, we further found that FK3006 belonged to ST15-KL112 isolates, a typical MDR clone. Moreover, 13 resistant elements, three resistant plasmids, and one virulence plasmid were detected in this isolate (Table 2). In addition, three key resistance genes played an important role in the acquisition of resistance to aminoglycoside antibiotics (*rmtF*), β-lactam antibiotics (*bla*_CTX-M-15_), and carbapenems (*bla*_OXA-232_). Moreover, rmpA2 as a critical factor activating the expression of capsular polysaccharides (CPS) genes which is responsible for CPS biosynthesis was detected in the IncFIB(K)/HI1B plasmid of FK3006. The *iucABCD*-*iutA* operon which encoding proteins necessary for aerobactin siderophore biosynthesis was also found in p3006-2.

**Table 2:** Several features of the FK3006 genome.

Parameter	p3006-2	p3006-3	p3006-7	p3006-11
Accension number	JANCTJ010000002	JANCTJ010000003	JANCTJ010000007	JANCTJ0100000011
Length(bp)	177,803	128,305	10,397	139,495
No. of ORF^a^	387	296	22	318
Incompatibility group	IncFIB(K)/HI1B	IncFIB(pKPHS1)	ColKP3	IncFII(K)
Conjugal ability	T4CP	NO	NO	T4CP
	oriT			T4SS
				oriT
				Relaxase
Resistance gene(s)	NO	*rmtF*	*bla*_OXA-232_	*sul2*
		*ARR-3*		*aph(3″)-Ib*
				*aph(6)-Id*
				*bla*_TEM-1B_
				*bla*_CTX-M-15_
				*dfrA14*
				*qnrB1*
Virulence factors	*rmpA2*	NO	NO	NO
	*iucABCD*			
	*iutA*			

^a^ORF, open reading frame.

### The non-conjugative pLVPK-like virulence plasmid could be transferred with the help of conjugative IncFII_K_ p3006-11 plasmid

**Figure 1:**
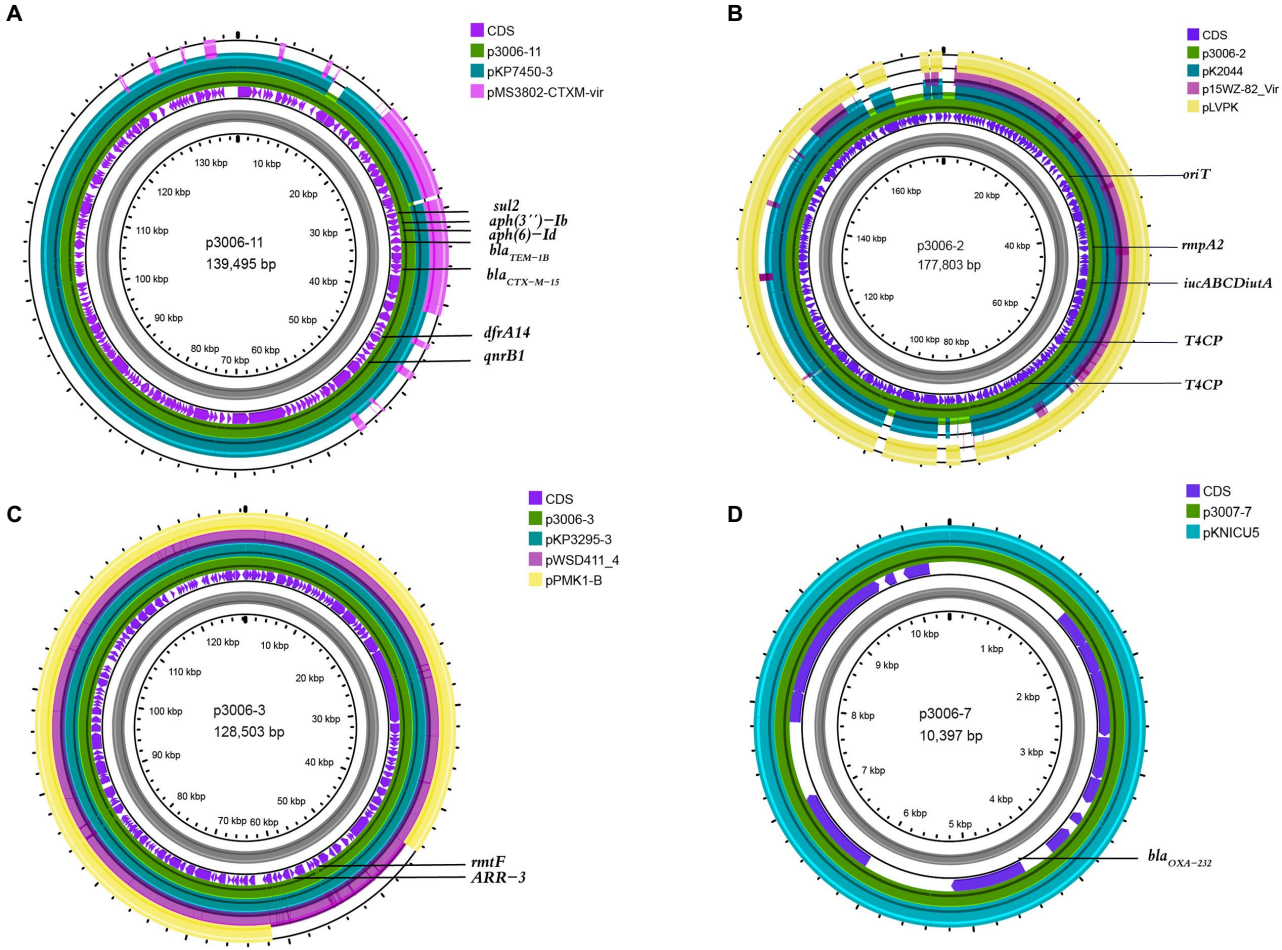
Comparative analysis of p3006-2, p3006-3, and p3006-7 plasmids with other reference plasmids. **(A)** Genome alignment was performed with p3006-11(JANCTJ010000011), pKP7450-3(99.95%, CP090471.1), and pMS3802-CTXM-vir(99.59%, CP068016.1). **(B)** Genome alignment was performed with p3006-2(JANCTJ010000002), virulent plasmid pK2044 (99.46%, CP026012.1), mobilisable virulence plasmid p15WZ-82-Vir(99.53%, CP032356.1), and pLVPK(99.51%, AY378100). **(C)** Genome alignment was performed with p3006-3(JANCTJ010000003), pKP3295-3(99.98%, CP079728.1), pWSD411_4(99.98%, CP045677.1), and mobilisable plasmid pPMK1-B(99.96%, CP008931.1). **(D)** Genome alignment was performed with p3006-7(JANCTJ010000007), and pKNICU5(KY454616.1).

**Figure 2:**
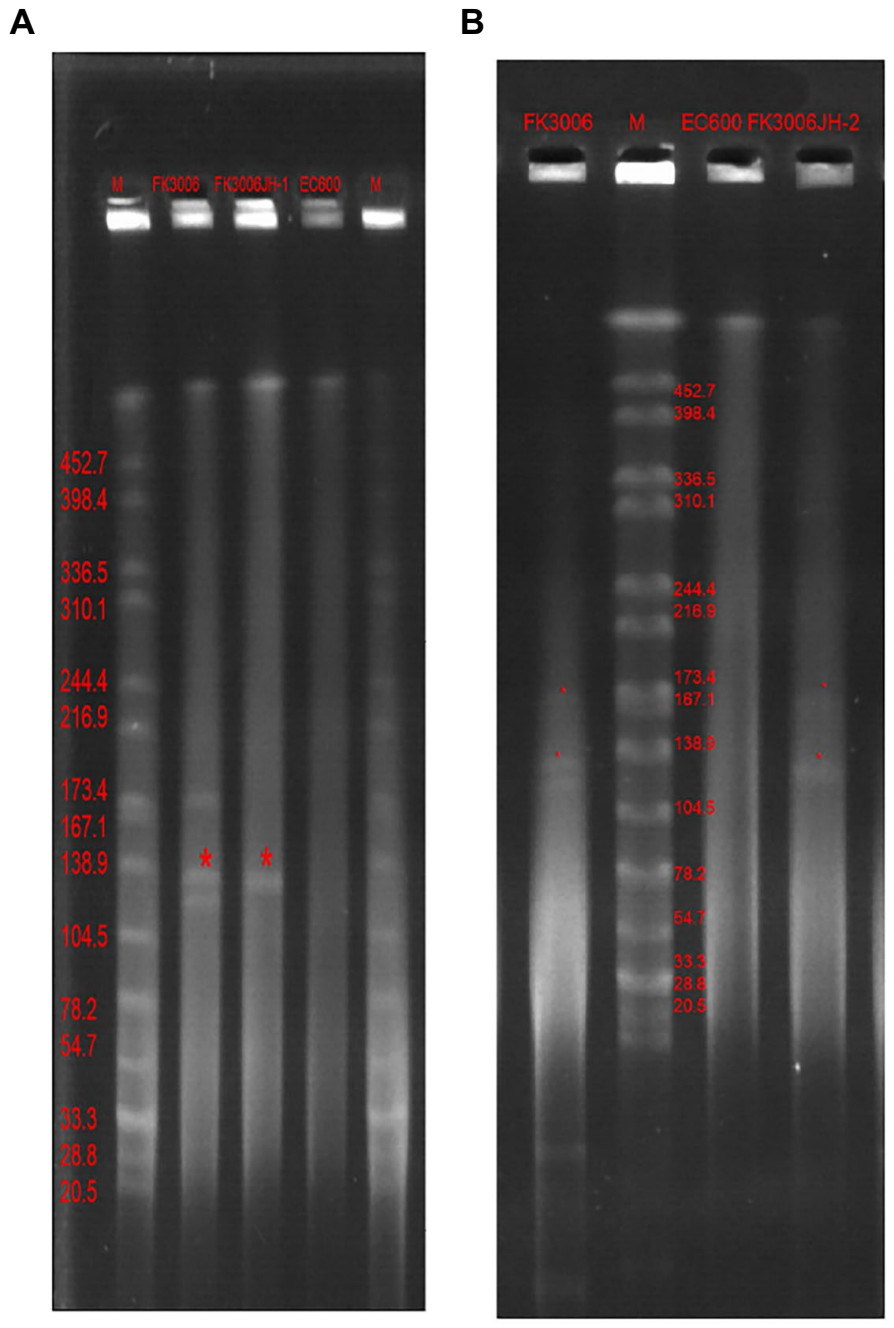
S1-PFGE profiles of FK3006, its transconjugants and *E.coli* 600 Lane marker was *X*baI digested genomic DNA from *Salmonella* Braenderup H9812. **(A)** The transconjugant(FK3006JH-1, the serial dilutions were plated on selective media with ampicillin, 100 μg/ml) only had one plasmid, with the size of ~139 kb(p3006-11, *bla*_*CTX*-M-15_). **(B)** The transconjugant (FK3006JH-2, the serial dilutions were plated on selective media with dipotassium tellurite, 8 μg/ml) had two plasmid, with the size of ~139 kb(p3006-11, *bla*_*CTX*-M-15_) and ~ 178 kb(p3006-2, *iucA*).

We have identified three key resistant plasmids, and one virulence plasmid in FK3006. As plasmids are often transmissible between bacteria, we made a detailed analysis of these plasmids, aiming to further clarify potential resistance and virulence dissemination threats of FK3006. In FK3006, we observed three resistant plasmids: p3006-3, p3006-7, and p3006-11. p3006-11 (*bla*_CTX-M-15_) was a typical IncFII_K_-type MDR plasmid and shared high identity with the mobilizable plasmid pKP7450-3(identity 99.95%, *bla*_CTX-M-15_, IncFII(K), CP090471.1), as well as the mobilizable plasmid pMS3802-CTXM-vir (identity 99.59%, *bla*_CTX-M-15_, IncHI1B(pNDM-MAR), CP068016.1; Figure 1A). p3006-11 harbored four conjugation modules, holding the potential to self-transfer. Hence, we applied conjugation assay to imitate and evaluate the dissemination ability of p3006-11 plasmid. We found the p3006-11 was successfully transferred from FK3006 to *E. coli* EC600 (1.1 × 10^−6^-9.7 × 10^−5^). Moreover, the S1-PFGE pattern (Figure 2A) and MICs of FK3006JH-1(EC600 harboring *bla*_CTX-M-15_ plasmid) also confirmed that the resistance phenotype dissemination of FK3006 (Table 1). Notably, the obtain of p3006-11 did not affect the growth of *E. coli* 600, which ensuring the stable existence of the resistance plasmid (Supplementary Figure S1).

p3006-2 was a typical IncFIB(K)/HI1B type virulence plasmid and shared ~99% identity with pK2044 (CP026012.1) and pLVPK (AY378100) plasmids (Figure 1B). Notably, p3006-2 also harbored the core *oriT* site and T4CP, which were identical to pK2044 and pLVPK. Previous studies have confirmed the pK2044 and pLVPK virulence plasmid could transferred from hypervirulent *K. pneumoniae* (hvKP) to ST11 CRKP and *E. coli* strains with the help of a self-transferable IncFII_K_ plasmid. In this study, we also observed that the p3006-2 virulence plasmid could be induced transfer with the help of p3006-11 plasmid (8.3 × 10^−8^-3.2 × 10^−7^; Figure 2B).

### Genetical features of other resistance plasmids

p3006-3 plasmid harbored *rmtF* gene and belonged to IncFIB type plasmid (Figure 1C), which was nearly identical (99.98%) to the human *K. pneumoniae* plasmid pKP3295-3 (*rmtF*, IncFIB(pKPHS1) CP079728.1) and pWSD411-4 (*rmtF*, IncFIB(pKPHS1), CP045677.1) previously reported in Hangzhou, China. Meanwhile, p3006-3 plasmid was similar to the pPMK1-B(coverage 86%, identity 99.96%, IncFIB(pKPHS1), CP008931.1) which also could be self-transferred to the recipient strain except for a multidrug resistance region of p3006-3 (Shi et al., 2020).

p3006-7 plasmid was a 10,397 bp circular molecule, harboring *bla*_OXA-232_ resistance element, and contained no necessary elements for transmission by bioinformatic analysis. The conjugation assay suggested that p3006-7 was not self-transferable. According to the genomic comparison, we found p3006-7 was almost identical to pKNICU5 plasmid (identity 99.93%, ColKP3, KY454616.1), isolated from the first OXA-232-producing *K. pneumoniae* strain in China (Figure 1D; Yin et al., 2017). As the self-transmissible modules were usually absent in such ColKP3 type plasmid, the *bla*_OXA-232_ genes may be mainly clonal dissemination.

### FK3006 was a typical hypervirulent strain and the virulence phenotype could be transferred to the recipients

**Figure 3:**
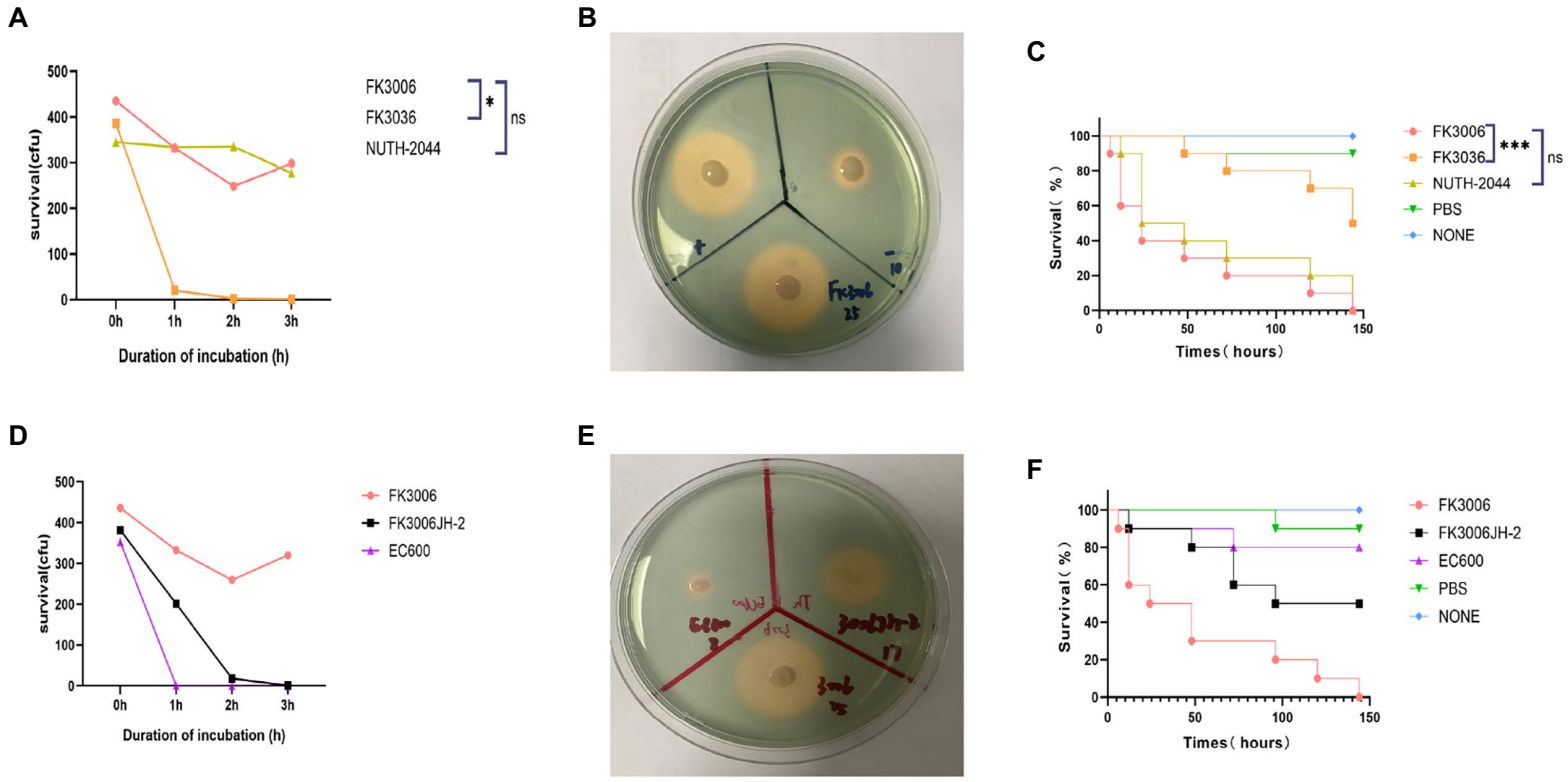
The virulence analysis of FK3006. **(A)** Serum killing assay of FK3006, FK3036 and NUTH-K2044 strains. Survival of each strain was evaluated by enumerating viable counts on MHA agar plate for 0, 1, 2, and 3 h of incubation in the pooled human serum at 37°C. There was a difference (*p* < 0.05) in the growth of the strain FK3006 and FK3036 *p < 0.05. **(B)** Siderophore production of FK3006, FK3036 and NTUH-K2044. FK3036 and NUTH-K2044 were used as negative and positive control, respectively. An orange halos zone indicated Siderophore production. **(C)** Survival rates of *G. mellonella* infected with FK3006, FK3036, NUTH-K2044, PBS, and none. Log-rank Mantel-638 Cox test was performed for analysis of the indicated curves. A difference (*p* < 0.001) was observed between FK3006 and FK3036 *** *p* < 0.001. **(D)** Serum killing assay of FK3006, FK3036JH-2 and EC600 strains. **(E)** Siderophore production of FK3006(25 mm), FK3006JH-2(17 mm) and EC600(8 mm). An orange halos zone indicated Siderophore production. **(F)** Survival rates of G. mellonella infected with FK3006, FK3006JH-2, EC600, PBS, and none.

The existence of pLVPK-like plasmid p3006-2 and the hypermucoviscosity indicated that the FK3006 may be a hypervirulent strain, here we applied several experiments to confirm the virulent phenotype. We found that FK3006 strain have a survival of about 74% after 60 min of incubation with the serum, which was significantly higher than that of the hypervirulence-negative resistant strain FK3036 (ST15 CRKP; Figure 3A). Moreover, there was a significant increase of siderophore production in FK3006 (*d* = 25 mm), compared with FK3036 (*d* = 10 mm), which did not have the *iuc* operon (Figure 3B). A549 human lung epithelial cells were infected with FK3006 (LDH, 1.70 μmol/l) led to cell LDH release of 76.9% compared with the positive control strain NUTH-K2044 group (LDH, 2.21 μmol/l). And, the negative control FK3036 group only release 0.85 μmol/l LDH. We also applied *G. mellonella* infection model to analyze the virulence of FK3006 *in vivo*. Testing showed that the survival rate of FK3006 group was 20% at 72 h, which was almost identical to that of a virulent strain of NUTH-K2044 (Figure 3C). A whole genome BLAST search was performed against VFDB to validate virulence factors harbored by the FK3006 (Supplementary Table S3). These results were in accordance with the fact that FK3006 populations were typical hypervirulent strains.

Notably, according to the conjugation assay, we observed the virulence plasmid p3006-2 could successfully transferred to the recipient strain EC600, and obtained the transconjugant FK3006JH-2. We found that FK3006JH-2(*iucA*) strains have a survival of about 53% after 60 min of incubation with the serum, which was significantly higher than that of the recipient strain EC600 (Figure 3D). When the EC600 obtained the p3006-2 plasmid, siderophore production also increased with the diameter of the halo increased ~2-fold (Figure 3E). *G. mellonella* infection model showed that the survival rate of FK3006JH-2 group was significantly lower than that of EC600 group (Figure 3F). All these results indicated that the virulence features of the FK3006 could be transferred to the recipients through the key virulence plasmid p3006-2.

### Mobile genetic elements associated with key resistance elements

**Figure 4:**
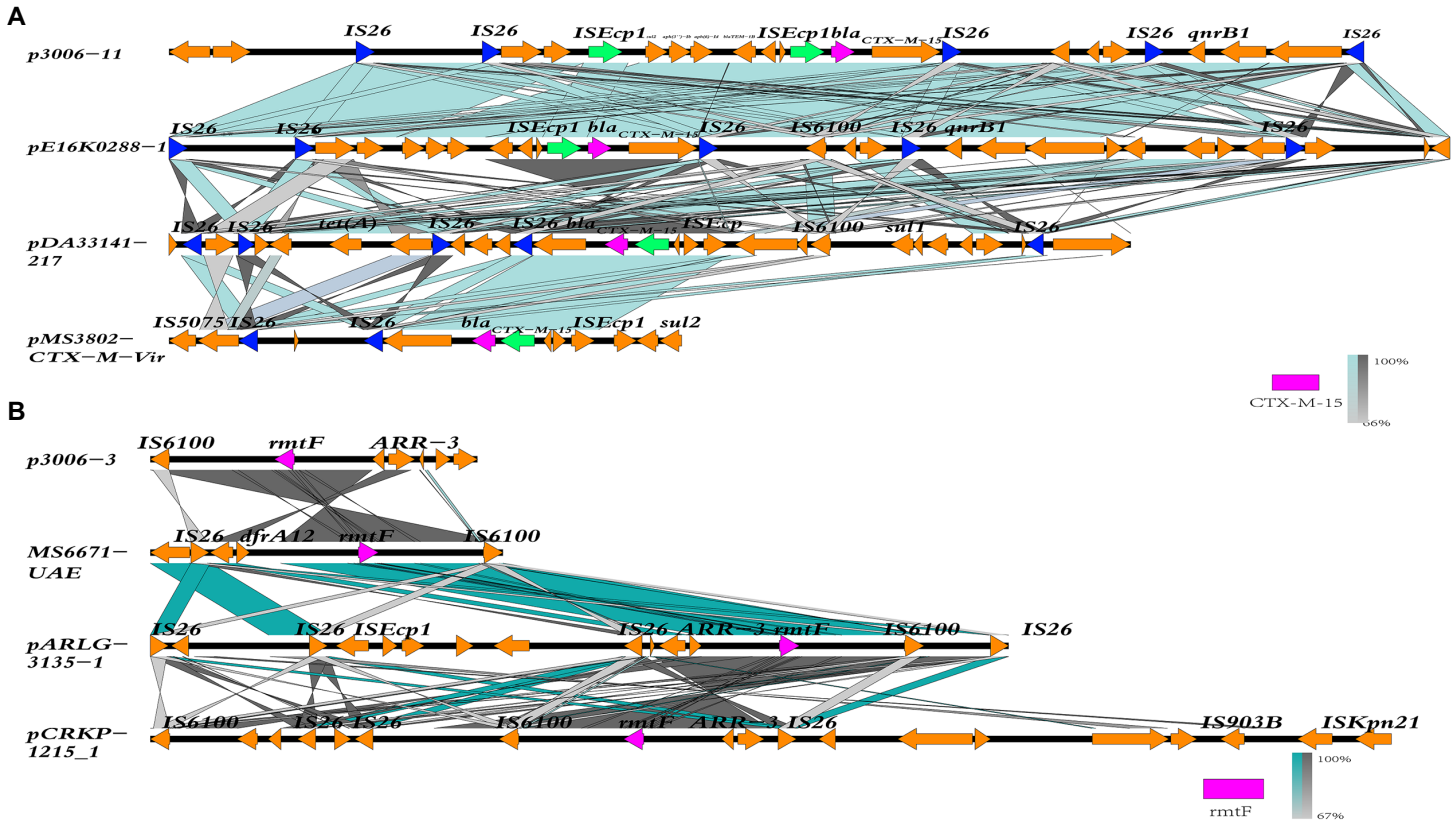
Linear comparison of the *bla*_*CTX*-M-15_ and *rmtF* region. **(A)** The *bla*_CTX-M-15_ region was compared with the regions extracted from pE16K0288-1(CP052263.1), pDA33141-217(CP029588.1), and pMS3802-CTX-M-Vir(NZ_CP068016.1). **(B)** The *rmtF* region was compared with the regions extracted from MS6671-UAE(LN824138.1), pARLG-3,135-1(CP033947.1), and pCRKP-1,215-1(NZ_CP024839.1).

The plasmid not only contains genes that promote the survival of the host but also carries other MGEs, such as Tn and IS, which also make a significant contribution to the dissemination of resistance genes. To thoroughly analyze the dissemination potential of *rmtF* and *bla*_CTX-M-15_ in FK3006, we also analyze each of the MGEs surrounding them. In p3006-11, the *bla*_CTX-M-15_, together with other antibiotic resistance genes (*sul2-aph(3″)-Ib-aph(6)-Id-blaTEM*_-1B-dfrA14_*- qnrB1*), was part of a large AMR of 37,526 bp that was bracketed by two opposite orientation copies of the IS*26* (Figure 4A) and it was closely associated with the presence of class 1 integrons. IS*Ecp1*, a member of the IS*1380* family frequently upstream of the *bla*_CTX-M-15_, seems to be a crucial element in the mobilization and dissemination of *bla*_CTX-M-15_, as it not only located surrounding *bla*_CTX-M-15_ in the p3006-11 plasmid, but also surrounding the *bla*_CTX-M-15_ in the pE16K0288-1(CP052263.1, IncFIB(K), IncFII(K), Korea), pDA33141-217(CP029588.1, IncFIB(K), IncFII(K), Sweden) and pMS3802-CTX-M-Vir(CP068016.1, IncHI1B(pNDM-MAR), repB, Spain) (Upadhyay et al., 2015; Fu et al., 2020; Hernandez et al., 2021). Similar to pCRKP-1,215_1 (CP024839.1, IncFII(pKPX1), Korea) and pARLG-3,135-1(CP033947.1, IncFIB(pQil), IncFII(K), United States), the typical IS*6100*-*rmtF*-*ARR-3* ARI was identified in p3006-3, containing class 1 integrons (Figure 4B). IS*6100*, like IS*26*, is a member of the IS*6* family, and has previously been described as the most common IS element adjacent to *rmtF* (Mataseje et al., 2014). However, most of IS*6100* appears in the vicinity of drug resistance genes alone unlike IS*26* (Partridge et al., 2018).

## Discussion

Globally, CRE including CRKP pose a major public health threat (Logan and Weinstein, 2017; Ernst et al., 2020). The notorious nosocomial pathogen hvKP exhibits enhanced virulence features and causes metastatic, and invasive infections (Russo and Marr, 2019). The phenotypes of MDR and hypervirulence in *K. pneumoniae* did not overlap for a long time as MDR phenotypes are often exhibited by classical *K. pneumoniae* (cKP) strains while the carriage of MDR genes in hvKP isolates was rare(Lee et al., 2017). However, more and more isolates with MDR and hypervirulence have been detected in the face of antibiotic selection pressure, this poses a wide array of problems for the treatment (Hennequin and Robin, 2016; Tang et al., 2020). According to epidemiological researches, most hv-CRKP were associated with *bla*_KPC-2_ and ST11 *K. pneumoniae*. However, in this study, we report an un-common co-existence of *bla*_OXA-232_, *rmtF,* and pLVPK-like virulence plasmid in a ST15 *K. pneumoniae*.

FK3006, which owned the typical hypermucoviscosity feature, was isolated from the sputum sample of a young patient admitted to the ICU direct postoperatively. In FK3006, we got three resistant plasmids: p3006-3 (*rmtF*, *ARR-3*), p3006-7(*bla*_OXA-232_) and p3006-11 (*bla*_CTX-M-15_), as well as a pLVPK-like virulence plasmid. The p3006-11 plasmid was conjugative and could be successfully self-transferred to EC600. Except for p3006-11 plasmid, other resistant plasmids were typed as non-mobile plasmid, as the core *oriT* site was absent in p3006-3 and p3006-7. Notably, although the p3006-2 virulence plasmid was typed as non-conjugative plasmid like pK2044 and pLVPK plasmid, we observed it could be induced mobilized with the help of p3006-11 plasmid as previously studied (Xu et al., 2021; Tian et al., 2022). The pLVPK-like virulence plasmids of *K. pneumoniae* are generally regarded as nonconjugative, these results indicated that the co-existence of IncFII_K_ resistant plasmid and pLVPK-like virulence plasmid would increase the risk for the virulence dissemination. Although previous studies have reported the co-existence of *bla*_OXA-232_ and virulence-like plasmid in a ST15 *K. pneumoniae*, such *isolate* did not exhibit the hypervirulent phenotype (Shu et al., 2019). However, in our study, the existence of pLVPK-like virulence plasmid endowed the typical hypervirulent characteristics to the FK3006, and such difference may be attributed to the expression variance or other unrecognized virulence determinants in FK3006.

The dissemination of resistance genes is not only *via* plasmids but also *via* other mobile structures. IS*Ecp1* was located in upstream of the *bla*_CTX-M-15_ gene, which was common in other reported *bla*_CTX-M-15_ elements. In addition, the promoter sequence-35(TTGAAA) and-10(TACAAT) regions in IS*Ecp1* provide a potential promoter for *bla*_CTX-M-15_ gene, inducing high expression of it (Ben Slama et al., 2011; Kieslich et al., 2016). Further, IS*26* surrounds *bla*_CTX-M-15_ gene and plays a key role in the dissemination (Seo and Lee, 2021). IS*26* can be located at the upstream of the CTX-M-15 gene, either alone or in combination with IS*Ecp1*. It was reported that IS*Ecp1* was often truncated when it co-existed with IS26, and the truncation position was not fixed. Notably, the promoter sequence of IS*Ecp1* was preserved (Diestra et al., 2009). In our study, IS*Ecp1* was not truncated by IS26. All in all, the surrounding environment of *bla*_CTX-M-15_ contains a variety of transposons and integrons. Meanwhile, coexisting with other drug-resistant genes in the same plasmid makes it easier for them to survive in the environment.

*Enterobacterales* isolates producing *rmtF* used to be extremely rare in China, but in recent years relevant reports have emerged and are always accompanied by coproduction of OXA-232. Plasmids with *rmtF* gene often acquired through multiple mobile elements. In this study, the *rmtF* plasmid p3006-3 could not self-transferred to other isolates, but the MGEs surrounding the *rmtF* generated a mobile ARI. The genetic background of the *rmtF* is associated with insertion sequence IS*6100*. Previous studies showed Tn*6229* was related to the Tn*3* family and carried a class 1 integron harboring the *rmtF* and IS*6100* (Mataseje et al., 2014). This ARI, together with several MGEs (IS*6100* and Tn*3*), can form a highly active transmission among the strains, which is extremely harmful.

In this study, we report the uncommon co-existence of *bla*_OXA-232_, *rmtF,* and a movable pLVPK-like virulence plasmid a ST15 *K. pneumoniae*. The association of antibiotic resistance genes with mobile genetic elements in FK3006 could promote rapid emergence of hv-CRKP strains. Notably, we found the co-exsistence of resistance and virulence plasmid not only generated the high-risk hypervirulent multidrug-resistant phenotype, but also increased the transmission threaten of non-conjugative virulence plasmid. In the future, when plasmid analysis becomes a routine detection method for such high-risk bacteria in medical institutions, necessary interventions could be carried out as early as possible, and the mortality rate might be reduced.

## Data availability statement

The datasets presented in this study can be found in online repositories. The names of the repository/repositories and accession number(s) can be found in the article/Supplementary material.

## Author contributions

CW: conceptualization, data curation, formal analysis, methodology, and writing–original draft. YZ: data curation, methodology, and writing–original draft. WA and HZ: methodology. YG, BW, and XnW: software. XaW: formal analysis. LR and JZ: writing–original draft. FY: conceptualization, project administration, and writing–review and editing. LW: conceptualization and writing–review and editing. All authors contributed to the article and approved the submitted version.

## Conflict of interest

The authors declare that the research was conducted in the absence of any commercial or financial relationships that could be construed as a potential conflict of interest.

## Publisher’s note

All claims expressed in this article are solely those of the authors and do not necessarily represent those of their affiliated organizations, or those of the publisher, the editors and the reviewers. Any product that may be evaluated in this article, or claim that may be made by its manufacturer, is not guaranteed or endorsed by the publisher.

## Footnotes

^1^ https://cge.cbs.dtu.dk/services/PlasmidFinder/

^2^ https://tool2-mml.sjtu.edu.cn/VRprofile/

^3^ https://tool-mml.sjtu.edu.cn/oriTfinder/oriTfinder.html

^4^ https://www-is.biotoul.fr/

^5^ https://proksee.ca/projects/10a9d9fa-c055-4dfe-b615-a55049475a52

^6^ http://www.mgc.ac.cn/cgi-bin/VFs/v5/main.cgi
