# Supplementary material for: Co-occurrence of OXA-232, RmtF-encoding plasmids, and pLVPK-like virulence plasmid contributed to the generation of ST15-KL112 hypervirulent multidrug-resistant Klebsiella pneumoniae
Source: Front Microbiol. 2023 Feb 28;14:1133590. doi: 10.3389/fmicb.2023.1133590 (PMC10011171; doi:10.3389/fmicb.2023.1133590)
Supplement: Supplementary file 1 [file Data_Sheet_1.docx]

Supplementary Material

Table S1 Information of FK3036 and NUTH-K2044

|  | Capsular serotype | MLST | Resistance genes | Virulence factors |
| --- | --- | --- | --- | --- |
| NUTH-K2044 | K1 | ST23 | NO | YES |
| FK3036 | KL24 | ST15 | YES | NO |

Table S2 Primers used in this study

| Name | Product size (bp) | Temperature (◦C) | Forward/reverse | Sequence |
| --- | --- | --- | --- | --- |
| *bla*_CTX-M-15_ | 581 | 58 | Forward | TTAGAGCGGCAGTCGGGAGG |
|  |  |  | reverse | ACCCAGGAAGCAGGCAGT |
| *iucA* | 555 | 59 | Forward | AGTCGGGAAGCGAGCCTGTA |
|  |  |  | reverse | AACTTTGGCGAGGCGGAGC |

Table S3 Virulence factors harbored by the FK3006

| **VFclass** | **Virulence factors** | **Related genes** | **FK3006(Prediction)**  **draft (draft)** |
| --- | --- | --- | --- |
| Adherence | Type 3 fimbriae | mrkA | 6666666.922396.peg.434 |
|  |  | mrkB | 6666666.922396.peg.435 |
|  |  | mrkC | 6666666.922396.peg.436 |
|  |  | mrkD | 6666666.922396.peg.437 |
|  |  | mrkF | 6666666.922396.peg.438 |
|  |  | mrkH | 6666666.922396.peg.441 |
|  |  | mrkI | 6666666.922396.peg.440 |
|  |  | mrkJ | 6666666.922396.peg.439 |
|  | Type I fimbriae | fimA | 6666666.922396.peg.195; 6666666.922396.peg.427 |
|  |  | fimB | 6666666.922396.peg.429 |
|  |  | fimC | 6666666.922396.peg.197; 6666666.922396.peg.425 |
|  |  | fimD | 6666666.922396.peg.198; 6666666.922396.peg.316; 6666666.922396.peg.424 |
|  |  | fimE | 6666666.922396.peg.428 |
|  |  | fimF | 6666666.922396.peg.201; 6666666.922396.peg.423 |
|  |  | fimG | 6666666.922396.peg.202; 6666666.922396.peg.422 |
|  |  | fimH | 6666666.922396.peg.203; 6666666.922396.peg.421 |
|  |  | fimI | 6666666.922396.peg.196; 6666666.922396.peg.426 |
|  |  | fimK | 6666666.922396.peg.204; 6666666.922396.peg.420 |
| Efflux pump | AcrAB | acrA | 6666666.922396.peg.3529 |
|  |  | acrB | 6666666.922396.peg.3; 6666666.922396.peg.3530 |
| Iron uptake | Aerobactin | iucA | 6666666.922396.peg.5280 |
|  |  | iucB | 6666666.922396.peg.5279 |
|  |  | iucC | 6666666.922396.peg.5278 |
|  |  | iucD | 6666666.922396.peg.5277 |
|  |  | iutA | 6666666.922396.peg.2839; 6666666.922396.peg.5276 |
|  | Ent siderophore | entA | 6666666.922396.peg.3351 |
|  |  | entB | 6666666.922396.peg.3352 |
|  |  | entC | 6666666.922396.peg.3354 |
|  |  | entD | 6666666.922396.peg.3364 |
|  |  | entE | 6666666.922396.peg.3353 |
|  |  | entF | 6666666.922396.peg.3360 |
|  |  | entS | 6666666.922396.peg.3356 |
|  |  | fepA | 6666666.922396.peg.3363; 6666666.922396.peg.4359 |
|  |  | fepB | 6666666.922396.peg.3355 |
|  |  | fepC | 6666666.922396.peg.3359 |
|  |  | fepD | 6666666.922396.peg.3357 |
|  |  | fepG | 6666666.922396.peg.3358 |
|  |  | fes | 6666666.922396.peg.3362 |
|  | Salmochelin | IroB | - |
|  |  | iroC | - |
|  |  | iroD | - |
|  |  | iroE | 6666666.922396.peg.2221 |
|  |  | iroN | 6666666.922396.peg.2651 |
|  | Yersiniabactin | fyuA | 6666666.922396.peg.1335 |
|  |  | irp1 | 6666666.922396.peg.1339 |
|  |  | irp2 | - |
|  |  | ybtA | 6666666.922396.peg.1342 |
|  |  | ybtE | 6666666.922396.peg.1336 |
|  |  | ybtP | 6666666.922396.peg.1343 |
|  |  | ybtQ | 6666666.922396.peg.1344 |
|  |  | ybtS | 6666666.922396.peg.1346 |
|  |  | ybtT | 6666666.922396.peg.1337 |
|  |  | ybtU | 6666666.922396.peg.1338 |
|  |  | ybtX | 6666666.922396.peg.1345 |
|  | Ferrousiron transport(Shigella) | sitB | 6666666.922396.peg.1368 |
|  |  | sitC | 6666666.922396.peg.1367 |
|  | Iron/manganese transport(Escherichia) | sitA | 6666666.922396.peg.1369 |
|  |  | sitD | 6666666.922396.peg.1366 |
| Nutritional factor | Allantoin utilization | allA | - |
|  |  | allB | - |
|  |  | allC | - |
|  |  | allD | - |
|  |  | allR | - |
|  |  | allS | - |
| Regulation | RcsAB | rcsA | 6666666.922396.peg.1409 |
|  |  | rcsB | 6666666.922396.peg.1080 |
|  | RmpA | rmpA | 6666666.922396.peg.5190 |
|  |  | clpV/tssH | 6666666.922396.peg.2535 |
|  |  | dotU/tssL | 6666666.922396.peg.2538 |
|  |  | hcp/tssD | 6666666.922396.peg.2536 |
|  |  | icmF/tssM | 6666666.922396.peg.2521 |
|  |  | impA/tssA | 6666666.922396.peg.2520 |
|  |  | ompA | 6666666.922396.peg.2537 |
|  |  | sciN/tssJ | 6666666.922396.peg.2514 |
|  |  | tle1 | - |
|  |  | tssF | 6666666.922396.peg.2516; 6666666.922396.peg.545 |
|  |  | tssG | 6666666.922396.peg.2515 |
|  |  | vasE/tssK | 6666666.922396.peg.2539 |
|  |  | vgrG/tssI | 6666666.922396.peg.2534 |
|  |  | vipA/tssB | 6666666.922396.peg.2541 |
|  |  | vipB/tssC | 6666666.922396.peg.2540 |
|  | T6SS-II | clpV | 6666666.922396.peg.797 |
|  |  | dotU | 6666666.922396.peg.552 |
|  |  | icmF | 6666666.922396.peg.546 |
|  |  | impF | 6666666.922396.peg.542 |
|  |  | impH | 6666666.922396.peg.544 |
|  |  | impJ | 6666666.922396.peg.553 |
|  |  | ompA | 6666666.922396.peg.551 |
|  |  | sciN | 6666666.922396.peg.543 |
|  |  | vasA/impG | - |
|  |  | vgrG | 6666666.922396.peg.550 |
|  | T6SS-III | - | 6666666.922396.peg.1568 |
|  |  | - | 6666666.922396.peg.1583 |
|  |  | - | 6666666.922396.peg.1584 |
|  |  | dotU | 6666666.922396.peg.1570 |
|  |  | icmF | 6666666.922396.peg.1577 |
|  |  | impA | 6666666.922396.peg.1582 |
|  |  | impF | 6666666.922396.peg.1581 |
|  |  | impG | 6666666.922396.peg.1578 |
|  |  | impH | 6666666.922396.peg.1579 |
|  |  | impJ | 6666666.922396.peg.1569 |
|  |  | lysM | - |
|  |  | ompA | 6666666.922396.peg.1571 |
|  |  | sciN | 6666666.922396.peg.1580 |
|  |  | vgrG | 6666666.922396.peg.1572 |
| Toxin | Colibactin | clbA | - |
|  |  | clbB | - |
|  |  | clbC | - |
|  |  | clbD | - |
|  |  | clbE | - |
|  |  | clbF | - |
|  |  | clbG | - |
|  |  | clbH | - |
|  |  | clbI | - |
|  |  | clbJ | - |
|  |  | clbK | - |
|  |  | clbL | - |
|  |  | clbM | - |
|  |  | clbN | - |
|  |  | clbO | - |
|  |  | clbP | - |
|  |  | clbQ | - |
|  |  | clbS | - |
| Stress adaptation | Manganese transport system(Neisseria) | mntB | 6666666.922396.peg.1378 |

-, no such information.
