# Supplementary figures and images for: Co-occurrence of OXA-232, RmtF-encoding plasmids, and pLVPK-like virulence plasmid contributed to the generation of ST15-KL112 hypervirulent multidrug-resistant Klebsiella pneumoniae
Source: Front Microbiol. 2023 Feb 28;14:1133590. doi: 10.3389/fmicb.2023.1133590 (PMC10011171; doi:10.3389/fmicb.2023.1133590)

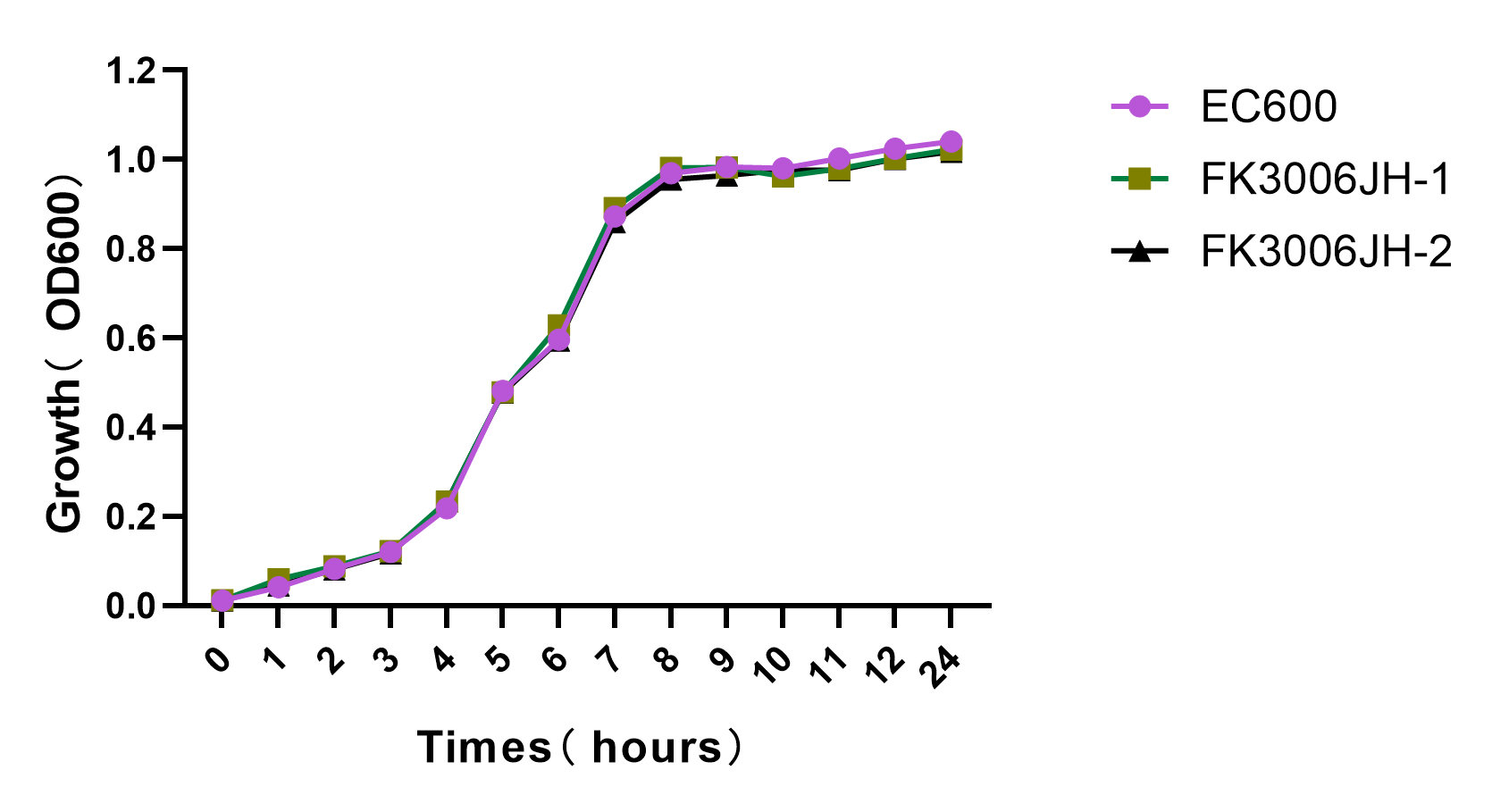

Supplement: Supplementary file 2 [file Image_1.TIF]
